# Development, validation, and reliability of a questionnaire to assess risk-factors of chronic kidney disease of unknown etiology

**DOI:** 10.1007/s40620-025-02297-3

**Published:** 2025-05-12

**Authors:** Asha K. Rajan, Nisha Abdul Khader, Pooja Gopal Poojari, Ravindra Prabhu Attur, Veena G. Kamath, Shobha U. Kamath, Shankar Prasad Nagaraju, Surulivel Rajan Mallayasamy, Muhammed Rashid, Girish Thunga

**Affiliations:** 1 Department of Pharmacy Practice, Manipal College of Pharmaceutical Sciences, Manipal Academy of Higher Education, 576104 Manipal, India; 2 Department of Nephrology, Kasturba Medical College, Manipal Academy of Higher Education, Manipal, 576104 India; 3 Department of Community Medicine, Kasturba Medical College, Manipal Academy of Higher Education, 576104 Manipal, India; 4 Department of Biochemistry, Kasturba Medical College, Manipal Academy of Higher Education, Manipal, 576104 India

**Keywords:** Chronic kidney disease of unknown aetiology, Agriculture, Pesticides, Drinking water, Heat stress

## Abstract

**Background:**

Chronic Kidney Disease of unknown aetiology (CKDu) is an emerging global health issue, yet research on risk factors is limited. This study addresses the gap by developing, validating, and evaluating the reliability of a questionnaire to assess the risk factors of CKDu.

**Methods:**

A draft version of the questionnaire was prepared based on thorough literature review and experts’ discussion. Six experts validated it for content validity, incorporating items with an item level content validity index ≥ 0.78. The reliability of the developed questionnaire was assessed using the test–retest method. Sample size for reliability was calculated using a tool by ArifinWN; 2018. Subjects were recruited by purposive sampling after obtaining informed consent. Data were analysed for reliability using Statistical-Package for the Social-Sciences (SPSS) version 20.0.

**Results:**

A 10-domain questionnaire was developed to assess CKDu risk factors. Six experts, comprised of three associate professors, two nephrologists and one renal pharmacist, validated the questionnaire. All items had an item level content validity index > 0.78, and the scale level content validity index of the questionnaire was 0.98. The questionnaire was administered to 165 subjects with a mean age of 50.65 ± 12.5 years, comprising 53.9% males. Most of them were labourers (38.2%) followed by farmers (21.8%). Among the risk factors, 57% of subjects were exposed to long hours of sunlight during work, 52.7% burned garbage waste and 46.1% were exposed to insecticides and pesticides. The questionnaire was re-administered after two weeks for reliability. All items under each domain fulfilled the minimum internal consistency of > 0.7.

**Conclusions:**

The questionnaire demonstrated its validity and reliability in assessing the risk factors of CKDu among the subjects.

**Graphical abstract:**

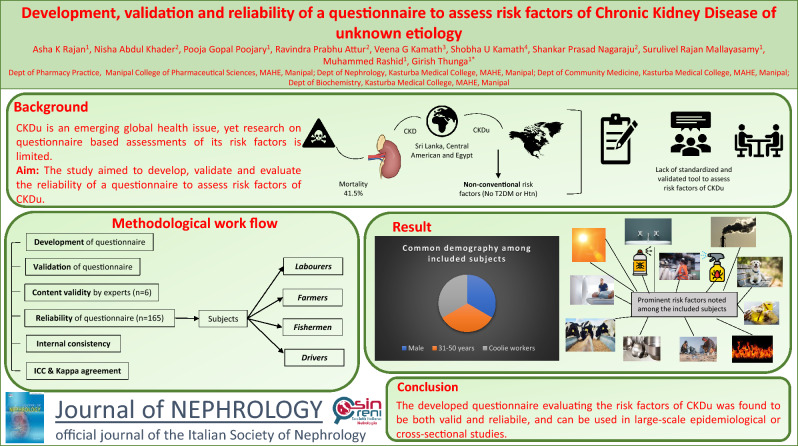

**Supplementary Information:**

The online version contains supplementary material available at 10.1007/s40620-025-02297-3.

## Introduction

Globally, chronic kidney disease (CKD) and kidney failure have emerged as significant public health challenges, leading to high rates of morbidity and mortality [1]. As per the report of global burden of disease, the mortality due to CKD has sharply increased [2]. Notably, the prevalence of CKD is escalating more rapidly in developing countries when compared to high income countries. Over the past two decades, reports from Sri Lanka[3], Central American countries, and Egypt have documented the emergence of a distinct form of severe CKD mainly affecting individuals in their fourth and fifth decades of life [4, 5]. This condition is unrelated to conventional risk factors such as diabetes or long-standing hypertension, and is termed as CKD of undefined aetiology (CKDu). There is growing concern that both CKD and CKDu are on the rise in India [6].

The exact cause(s) of CKDu remains unknown, with potential factors including heat exposure [7], chemical exposure, exposure to contaminated water (including heavy metals) [8], infections and various other environmental factors [9]. The global prevalence of CKDu is estimated to be approximately 11–13% among CKD patients, with a notable increase observed in lower-middle income and developing countries [10]. Initially, CKDu often remains undiagnosed until it reaches advanced stages, and results in poor prognosis [11]. Currently, there are no identified risk factors to predict disease onset and progression [9]. This rising health concern is predominantly high in the Middle East [12], Africa [13, 14], Asia [15], and Central America regions [16, 17]. Despite ongoing research, the exact aetiology remains unknown [18]. Researchers have highlighted six primary elements suspected of contributing to CKDu: exposure to heavy metals, exposure to pesticides, heat stress, strenuous labour, dehydration, and contamination of water and agricultural lands [10].

CKDu is disproportionately affecting marginalised labour and farming communities in tropical nations, where physical exertion is a part of daily life [10]. Interventions such as reducing heat exposure and promoting adequate hydration have shown promising benefits [7, 19]. Despite ongoing debate, there remains a paucity of published research on specific environmental risk factors for CKDu in India [20]. Several hypotheses have been proposed, including elevated concentrations of silica and heavy metals in water sources, prolonged periods of dehydration, heat stress, use of nonsteroidal anti-inflammatory drugs, use of traditional remedies, and excessive use of pesticides. However, conclusive evidence is lacking [21, 22]. Occupational heat exposure and dehydration are widely recognised as potential factors contributing to the aetiology and/or progression of CKDu [23].

Despite the significant impact of CKDu, upstream factors have been largely overlooked, partly due to the delayed detection and treatment of CKD [20]. Investigating the relationship between environmental factors and CKDu is crucial. Epidemiological data remain scarce, particularly within India [24, 25]. Longitudinal studies are essential to enhance understanding of critical disease risk factors and inform the development of preventive policies [20]. To date, causative factors, risk determinants, or early diagnostic techniques for CKDu remain elusive, leaving the disease’s origin a mystery [10]. Therefore, exploring individual’s perceptions of CKDu development using a standardised and validated tool tailored to the country’s context could offer insights into the most important risk factors in these populations.

In social science research, questionnaires have been widely utilised as a practical and affordable method to measure individual perception of the risk of developing specific diseases [26]. Therefore, the objective of the current study was to develop, validate, and assess the reliability of a comprehensive, user-friendly questionnaire to evaluate the risk factors associated with CKDu. This questionnaire also aims to provide a better understanding to healthcare providers, including primary care physicians, nurses, pharmacists, anganwadi workers (people employed to provide additional and supplementary healthcare and nutritional services to children and pregnant women), and accredited social health activists (ASHA workers) regarding the risk factors associated with CKDu.

## Materials and methods

### Ethical clearance

**Fig. 1:**
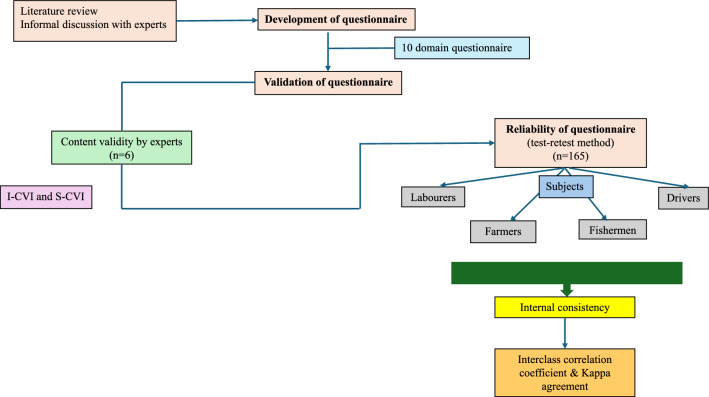
Workflow of validation and reliability of the questionnaire

Institutional ethical clearance was obtained before initiating the study (IEC:326/2020) and the work was carried out in accordance with the Declaration of Helsinki. The study is also registered in clinical trial registry of India (CTRI/2020/10/028729). Figure 1 provides a description of the study methodology.

### Development of the questionnaire

By referring to previously published literature and after informal discussion with experts, the draft version of the questionnaire consisting of ten distinct domains was prepared in English. Part I of the questionnaire included personal information such as demographic details, current and previous occupation, family history, medical and medication history, and social history of the subjects. Part II focused on whether the subjects already had any known risk factors for CKD. This included details on any previous history of kidney disease, risk factors for kidney disease, urine infections or urine tests in the past, type of treatment generally preferred for mild ailments. Part III was on the use of agrochemicals, which included chemical fertilisers, pesticides, insecticides, or anti-mite products for pets at home. Occupational habits of the subjects such as duration of work, intake of fluids and frequency of urination during working hours were included in part IV. Part V was on the use of over-the-counter medications. Information on drinking water, which included source, mixing of ground water with well water, usage of water purifiers, nearby industries, and sources of drinking water for cattle and poultry farming were included in part VI. Heat stress was another focused area in literature for which we included questions on duration of sunlight exposure during work and use of protection from the sun in part VII. Part VIII of the questionnaire was on air pollution, focusing on burning of crops and plastic waste, sources of fuel for cooking and types of cookware used. History of worm infestations, its management and deworming were included in part IX. Finally, part X included food and nutrition, consisting of preservation of food, intake of soda and soft drinks, skipping meals and intake of commercially prepared or restaurant food. The questionnaire was administered in Kannada, the local language spoken by the study participants, and back translated into English to ensure clarity and ease of comprehension.

## Validation of the questionnaire

### Content validity of the questionnaire

The developed questionnaire was validated by an expert team of six members. This included three associate professors from the department of pharmacy practice, two nephrologists and one renal clinical pharmacist, all of whom were invited to validate the questionnaire after obtaining informed consent from the participants. The suggested changes were incorporated into the final questionnaire after discussion with the team members and other expert panel members.

Content validity of the developed questionnaire was carried out to ensure appropriateness, accuracy, and relevance of the items separately. A 4-point Likert scale (not relevant, somewhat relevant, relevant, and highly relevant) was used for validating each item [27]. The developed questionnaire was validated by using the item content validity index and scale content validity index. Individual item content validity was determined by the item level content validity index, and whole questionnaire content validity was determined by the scale level content validity index which should be > 0.80 [28].

### Reliability of the questionnaire

Reliability of the developed questionnaire was assessed using the test–retest method. Assessment of reliability was performed through internal consistency, which is a frequently employed method to evaluate the dependability of an instrument. Internal consistency and reliability serve the purpose of examining the alignment and proximity of item responses, or a group of items, within a specific domain [29]. The evaluation of internal consistency for reliability analysis involved internal consistency using inter-item correlation. Since most of the items within the domains were categorical variables, the reliability coefficients (interclass correlation and kappa) were also determined.

### Sample size calculation

Sample size for test–retest reliability of the questionnaire was calculated using a tool developed by Arifin WN; 2018 [30]. The interclass correlation coefficient hypothesis testing calculator was used with the following values (minimum acceptable reliability- 0.70, expected reliability-0.80, significance level- 0.05 two tailed, power- 80%, number of raters- 2, expected drop out- 10%). The minimum acceptable reliability for this study was set at 0.70, which is a widely accepted threshold in health-related research indicating adequate reliability for questionnaires [31]. We selected an expected reliability value of 0.80 based on previous studies that have validated similar health risk assessment tools [32]. The significance level was established at 0.05 (two-tailed), a standard alpha level in medical research that balances Type I and Type II errors, while an 80% power was chosen as it is commonly accepted in research to detect true effects without increasing the risk of false positives. Additionally, we accounted for two raters and anticipated a 10% dropout rate based on our study design and prior experiences with similar populations. The calculated sample size of 148 was increased to 165 to accommodate potential dropouts.

### Study population and recruitment strategy

The study recruited subjects using a purposive sampling method to ensure the inclusion of individuals at potential risk of CKDu. Participants were selected from rural and semi-urban regions of Udupi district of Karnataka, South India, where CKDu has been reported. Subjects aged ≥ 18 years involved in occupations with high physical exertion, such as farming, fishing, driving, construction, as well as daily wage labourers, who were willing to provide written informed consent were included in the study after explaining the participant information sheet. Those with a prior history of CKD, or other chronic illnesses, who gave only test but not retest response were excluded to maintain focus on identifying potential risk factors among the general working population.

### Community-based approach

The study was conducted in the community setting, with field visits undertaken in multiple geographical locations. Data collection was carried out through face-to-face structured interviews at primary healthcare centres, community meeting areas and participants’ residence, ensuring comprehensive participation.

### Selection of study locations

The four study locations (I–IV) represent different rural clusters of Udupi district (Alevoor, Kadekar, Kaup, Malpe). These locations were selected based on epidemiological data, community healthcare reports, and accessibility for study implementation. The selection aimed to include variability in environmental and occupational exposures.

### Interviewer training

The research team implemented a comprehensive training program for all interviewers prior to data collection. The process encompassed several key components to ensure high-quality data gathering. Interviewers underwent an in-depth review of the questionnaire’s content and objectives, ensuring a thorough understanding of each item’s purpose and significance. The training focused on standardised interview methodologies to ensure consistent administration across all participants. To refine interviewing skills, hands-on practice sessions with mock interviews, followed by constructive feedback was done. Interviewers received guidance on maintaining neutrality and avoiding leading questions to prevent bias in participant responses. To facilitate effective communication, they were trained in the local language and collaborated with ASHA workers during field visits.

### Questionnaire administration

The questionnaire was administered by trained health professionals, including members of the research team and accredited social health activists. These individuals were trained in standardised interviewing techniques to minimise bias and ensure clarity in responses. Interviews were conducted in Kannada (the local language) to facilitate better comprehension.

### Participant characteristics and enrolment

The study included asymptomatic healthy individuals who were screened for CKDu. The goal was to assess potential environmental and occupational risk factors contributing to CKDu development. The uniform data collection strategy comprised introducing the study and obtaining informed consent, systematically presenting questions and recording responses, and providing clarification when participants required assistance. Throughout the data collection process, regular quality checks were conducted to maintain high standards of data integrity. Prior to participant enrolment, the research team provided a detailed explanation of the study, and informed consent was obtained only after ensuring participants’ understanding. The questionnaires were administered in the local language by the investigator, employing a purposive sample method to select participants.

### Interview

Interviews were conducted in private settings to ensure confidentiality, creating a safe environment where participants felt more comfortable sharing honest responses without fear of judgement or repercussions. Questions were carefully phrased using neutral language to avoid implying judgement, reducing the likelihood of leading participants to socially desirable answers and encouraging more genuine responses. For the most sensitive questions, participants were given the option to complete those sections privately. Participants were repeatedly assured of the confidentiality of their responses, helping to build trust and encourage more truthful answers. For some sensitive topics, indirect questioning techniques were employed. These methods can help participants feel more comfortable providing information about sensitive situations.

### Follow-up

After a period of two weeks, the same questionnaire was administered to the same subjects to check for reliability. A repeated test after a two-week time period is optimal to avoid the likelihood of participants remembering their previous responses, and it enhances better follow-up of the same population, timely data collection, as well as minimising the potential logistic challenges.

### Statistical analyses

Data analysis was conducted using IBM’s Statistical Package for the Social Sciences (SPSS) version 20.0 [33]. Mean ± SD was used to report continuous variables, and frequency with percentages were used to report categorical variables. The stability of response was assessed using test–retest reliability. The questionnaire’s reliability was assessed using Cohen’s Kappa statistics to each item within the 10-domains. The Kappa range of 0.00–0.20 indicates poor agreement, 0.21–0.40 signifies fair agreement, 0.41–0.60 represents moderate agreement, 0.61–0.80 indicates good agreement and 0.81–1.00 signifies very good agreement for categorical variables [34]. The chosen cut-off value of kappa agreement was 0.7 for this study [31, 35], as we aimed to ensure a level of agreement that falls within the upper range of “moderate” agreement and approaches “strong” agreement. Intra-rater reliability with absolute agreement using the interclass correlation was utilised to assess the reliability of all items. Interclass correlation values falling within the range of 0.60–0.74 were considered good, while those exceeding 0.75 were considered excellent [36]. Cronbach’s alpha was not applicable due to the questionnaire’s multidimensional structure and data type.

## Results

### Content validity

The questionnaire was validated by six experts and the item level content validity index was assessed for accuracy, appropriateness, and relevance. All items in the questionnaire except for “marital status” and “working habit of the participants” achieved an item level content validity index of > 0.78. These two items, belonging to the demographic and occupation domains, had a lower item level content validity index of 0.6. We decided to retain these items based on their theoretical importance as per the literature in CKDu research [37, 38], expert qualitative feedback emphasising their potential significance, precedent in literature and the exploratory nature of our study.

Ultimately, the final questionnaire was comprised of 64 items. The scale level content validity index value for the entire questionnaire was 0.98 for accuracy, appropriateness, and relevance. Detailed information on content validity is presented in Tables 3a–c, which correspond to accuracy, appropriateness, and relevance, respectively.

### Demographic characteristics

The questionnaire was administered to a total of 165 subjects. The average age of the study population was 50.65 ± 12.5 years. Most individuals were in the 31–50 year age group [74 (44.8%)]. Most of the subjects were male [89 (53.9%)], literate [138 (83.6%)], the majority of whom completed primary school education; III-VIII standard [73 (44.2%)]. The majority were labourers [63 (38.2%)] followed by farmers [36 (21.8%)]. The demographic details of the included subjects are given in Table 1.

**Table 1:** Demographic details of the subjects

Demographics	N (%)
Age	50.65 ± 12.5 years
18–30	8 (4.8)
31–50	74 (44.8)
51–70	66 (40)
> 71	17 (10.3)
Gender	
Male	89 (53.9)
Female	76 (46.1)
Location	
I (Alevoor)	100 (60.6)
II (Kadekar)	41 (24.8)
III (Kaup)	10 (6.1)
IV (Malpe)	14 (8.5)
Education	
Literate	138 (83.6)
Illiterate	27 (16.4)
Occupation	
Farmer	40 (24.2)
Driver	34 (20.6)
Labourer	67 (40.6)
Fishing	24 (14.5)

Various risk factors for CKDu were assessed. A total of 31 (18.8%) subjects had a history of anaemia and 74 (44.8%) participants reported prior radiation exposure, primarily from repeated medical imaging (eg: X-rays, CT scans) and to a lesser extent, occupational exposure in industrial settings [45 (27.3%) participants]. A considerable number of subjects [76 (46.1%)] were exposed to insecticides used for crops and on their home premises. A total of 119 (72.1%) subjects had pets at home. The majority of subjects [94 (57%)] were exposed to sunlight. Eighty-seven (52.7%) subjects within the population preferred “burning” to dispose of their household waste. Deworming, defined as the use of antiparasitic medications such as albendazole or mebendazole [39], was reported by 27.9% of participants. The lack of regular deworming in the remaining participants may indicate poor sanitation and chronic exposure to parasitic infections, which could contribute to systemic inflammation and potential renal insult. The details on risk factors among the included subjects are provided in Table 2.

**Table 2:** Risk factors

Risk factors	N (%)
Social history	
Yes	80 (48.5)
No	85 (51.5)
History of anaemia	
Yes	31 (18.8)
No	134 (81.2)
Bone or joint pains	
Yes	57 (34.5)
No	108 (65.5)
Issues during urination	
Yes	16 (9.7)
No	149 (90.3)
Urinary infection in the past	
Yes	16 (9.7)
No	149 (90.3)
Renal calculus in the past	
Yes	22 (13.3)
No	143 (86.7)
Exposure to scans or radiation	
Yes	74 (44.8)
No	91 (55.2)
Type of medicine generally used	
Allopathic	127 (77)
Ayurvedic	27 (16.4)
Folk medicine	2 (1.2)
Mixed	9 (5.5)
Cultivate vegetables and fruits	
Yes	71 (43)
No	94 (57)
Pesticides for crops	
Yes	49 (29.7)
No	116 (70.3)
Insecticides for crops and at home	
Yes	76 (46.1)
No	89 (53.9)
Pets at home	
Yes	119 (72.1)
No	46 (27.9)
Number of working days in a week	5.88 ± 1.5 days
Number of working hours in a day	7.29 ± 3.7 h
Home remedies for general ailments	
Yes	71 (43)
No	94 (57)
Source of drinking water	
Own well water	89 (53.9)
Bore well water	5 (3)
Panchayat (village council) water	35 (21.2)
Neighbours’ well water	26 (15.8)
Mixed	10 (6.1)
Cleaning of well	
Yes	50 (30.3)
No	50 (30.3)
NA	65 (39.4)
Cattle and poultry farming	
Yes	66 (40)
No	99 (60)
Exposure to sunlight during work	
Yes	94 (57)
No	71 (43)
Protection from heat	
Yes	45 (27.3)
No	120 (72.7)
Disposal of waste	
Burning	87 (52.7)
Municipality	55 (33.3)
Dumping nearby river	6 (3.6)
Composite	3 (8.5)
Mixed	14 (1.8)
Source of gas	
LPG	27 (16.4)
LPG/Traditional	127 (77)
Traditional	7 (4.2)
Multiple techniques	4 (2.4)
Practice of deworming	
Yes	46 (27.9)
No	119 (72.1)
Soda or soft drinks	
Yes	62 (37.6)
No	103 (62.4)
Frequency of eating commercially prepared or restaurant food	
Very rare	96 (58.2)
Once in a month	15 (9.1)
Once in a week	19 (11.5)
Everyday	35 (21.2)
Habit of skipping meals	
Yes	14 (8.5)
No	151 (91.5)

### Reliability

The questionnaire’s reliability was evaluated by the test–retest method. The questionnaire was readministered after a period of two weeks to check its reliability. Most of the items under each domain had an interclass correlation of 1.00. Range of interclass correlation coefficient was high for the domains of over-the-counter medications [1.00], agrochemicals [0.98–1.00], occupation [0.98–1.00], heat stress [0.98–0.99], and worm infestations [0.98–1.00].

Similarly, kappa values were high for the domain of over-the-counter medications (1.00), worm infestations (0.97–1.00), heat stress (0.97–0.98), occupation (0.93–1.00) and agrochemicals (0.96–1.00). Each item within every domain met the minimum internal consistency reliability threshold of 0.7, which is considered suitable for such exploratory research. Table 3 displays the reliability outcomes for each item.

**Table 3:** Results of the test–retest reliability of hard workers to assess the risk of CKDu: Interclass correlation coefficient (ICC), kappa and percentage agreement (item-specific)

Item (per part of the questionnaire)	Test–retest reliability			Usability
	ICC (95% CI)	Kappa	Agree	
1. Personal information				
Previous occupation	1.00 (1.00–1.00)	/	100.0	√
Family history of kidney disease	0.93 (0.91–0.95)	/	87.5	√
Family history other than kidney disease	0.97 (0.96–0.98)	/	94.9	√
Past medical history	0.99 (0.98–0.99)	/	98.7	√
Past medication history	0.99 (0.98–0.99)	/	98.7	√
Social history	0.96 (0.94–0.97)	/	92.7	√
2. Past health issues				
History of kidney problems at birth	1.00 (1.00–1.00)	/	100.0	√
Diagnosis of kidney disease	0.93 (0.91–0.95)	/	86.7	√
History of anaemia or low haemoglobin levels	0.99 (0.98–0.99)	/	98.0	√
Bone or joint pain	0.98 (0.97–0.98)	/	96.0	√
Issues during urination in the past	1.00 (1.00–1.00)	/	100.0	√
Urine test past 1 year	0.98 (0.97–0.98)	/	96.8	√
Urinary infections in the past	0.96 (0.95–0.97)	/	93.4	√
Renal calculus	1.00 (1.00–1.00)	/	100.0	√
Exposure to scan or radiation	0.98 (0.98–0.99)	/	97.5	√
Swelling of face and legs	1.00 (1.00–1.00)	/	100.0	√
Type of medicine generally used	1.00 (1.00–1.00)	/	100.0	√
Vitamin supplements used without doctor’s advice	0.98 (0.97–0.98)	/	100.0	√
Salt restricted diet advice from healthcare provider	1.00 (1.00–1.00)	/	100.0	√
Fluid restricted diet from healthcare provider	1.00 (1.00–1.00)	/	100.0	√
3. Agrochemicals				
Cultivate vegetables and fruits	0.99 (0.98–0.99)	/	98.8	√
Pesticides for cultivation	0.99 (0.98–0.99)	/	98.6	√
Using protection while using fertilisers	0.98 (0.97–0.98)	/	96.4	√
Storing pesticides at home premises	1.00 (1.00–1.00)	/	100.0	√
Insecticides for mosquitoes and mites at home	1.00 (1.00–1.00)	/	100.0	√
Pets at home	1.00 (1.00–1.00)	/	100.0	√
Washing hands after returning home	1.00 (1.00–1.00)	/	100.0	√
4. Occupation				
Number of working days in a week	0.99 (0.98–0.99)	/	95.5	√
Number of working hours in a day	1.00 (0.99–1.00)	/	97.2	√
Type of work	1.00 (1.00–1.00)	/	100.0	√
Quantity of hydration	0.98 (0.98–0.99)	/	93.5	√
Frequency of urination	0.99 (0.98–0.99)	/	93.7	√
5. OTC Medications				
Self-medicating for minor ailments	1.00 (1.00–1.00)	/	100.0	√
Home remedies for general ailments	1.00 (1.00–1.00)	/	100.0	√
Self-medicating on pain medications	1.00 (1.00–1.00)	/	100.0	√
6. Drinking water				
Source of drinking water	1.00 (1.00–1.00)	/	100.0	√
Cleaning of well	0.99 (0.98–0.99)	/	98.2	√
Well water mixing with ground water	0.95 (0.92–0.93)	/	90.4	√
Water purifier for drinking water	1.00 (1.00–1.00)	/	100.0	√
Non-availability of purifier	1.00 (1.00–1.00)	/	100.0	√
Nearby industries	0.93 (0.91–0.95)	/	87.6	√
Cattle and poultry farming	0.99 (0.98–0.99)	/	98.7	√
7. Heat stress				
Exposure to sunlight	0.98 (0.98–0.99)	/	97.5	√
Protection from heat	0.99 (0.98–0.99)	/	98.8	√
8. Air pollution				
Disposal of waste	1.00 (1.00–1.00)	/	100.0	√
Source of gas	1.00 (1.00–1.00)	/	100.0	√
Traditional ways of cooking	1.00 (1.00–1.00)	/	100.0	√
Cooking in steel vessels	0.93 (0.91–0.95)	/	87.5	√
Cooking in mud vessels	0.99 (0.98–0.99)	/	98.5	√
Cooking in aluminium vessels	0.98 (0.97–0.98)	/	96.0	√
Cooking in copper vessels	1.00 (1.00–1.00)	/	100.0	√
Cooking in non-stick vessels	0.99 (0.98–0.99)	/	98.6	√
Cooking in iron vessels	0.97 (0.96–0.97)	/	94.3	√
9. Worm infestations				
Practice of deworming	0.98 (0.97–0.98)	/	97.0	√
History of worm infestations	1.00 (1.00–1.00)	/	100.0	√
Management for worm infestations	0.99 (0.98–0.99)	/	98.5	√
10. Food and nutrition				
Diet	0.97 (0.96–0.98)	/	89.5	√
Preserve food at home	1.00 (1.00–1.00)	/	100.0	√
Storing food or water for long term in aluminium	0.88 (0.84–0.91)	/	79.7	√
Storing food or water for long term in mud	0.98 (0.98–0.99)	/	97.2	√
Storing food or water for long term in steel	0.98 (0.97–0.98)	/	96.1	√
Consuming soft drinks or soda	0.99 (0.98–0.99)	/	98.7	√
Consume tea or coffee	0.99 (0.98–0.99)	/	95.2	√
Frequency of eating commercially prepared or restaurant food	0.99 (0.98–0.99)	/	98.0	√
Habit of skipping meals	0.96 (94.9–97.2)	/	92.7	√

## Discussion

Globally, there has been an increase in the incidence of CKDu whose risk factors vary by region, cultural habits, and occupation [40]. The questionnaire developed in this study focused on identifying specific risk factors for CKDu, such as agrochemicals, drinking water, heat stress, air pollution, food & nutrition, occupation, over-the-counter products and worm infestations. The questionnaire was thoroughly validated with experts assessing its content; it was also tested for reliability in terms of inter-class correlation and kappa’s agreement.

The expert group, consisting of six members, reviewed the questionnaire and reached a consensus for adequate inter-rater agreement [41]. It is crucial for all the items to align with appropriateness, accuracy and relevance, since the assessment is meant to evaluate each item [42, 43]. The questionnaire consisted of ten domains which were further split into 64 individual items.

A two-week interval between test administrations was selected based on expert discussion and also on the basis of previous studies validating health-related questionnaires which have successfully used similar intervals [44–46]. Reliability (internal consistency) of our questionnaire was confirmed through interclass correlation (95% CI) showing a strong correlation among different factors within each domain, with all correlation values > 0.7, which is considered acceptable. The reliability scores aligned with recommended validated guidelines [47]. The questionnaire had a very good reliability and validity and could be utilised to evaluate the risk factors of CKDu. The reliability score was also consistent with the reported studies in the literature [48].

The degree to which a study’s validity and reliability concerns have been thoroughly examined is a crucial factor in research evaluation and influences the choice of whether to incorporate the study’s conclusions into practice. Rigour in qualitative research is ascertained by assessing the reliability and validity of the instruments or tools employed in the study [49].

From a public health standpoint, identifying the factors which contribute to a disease condition is very important for effective disease management [50]. Furthermore, it is advisable to provide targeted education on health literacy and knowledge enhancement. This would help in improving disease-specific insights concerning recommended daily practices and occupational habits. The developed questionnaire was comprehensive, as we tried to include maximum domains of risk factors of CKDu. As the experts evaluated the content validity, they ensured that the tool encompasses numerous items demanding a thorough understanding of the risk factors of the disease. The questionnaire was designed for a specific geographical region, and its applicability to other populations may require adaptation based on cultural, occupational and healthcare system differences. Future studies should validate the tool in diverse settings to ensure its broader applicability. Despite our efforts to mitigate bias, some degree of social desirability bias may still be present in our data, particularly for sensitive questions. This could potentially lead to underreporting of behaviours like alcohol consumption or smoking. However, the consistent application of our bias mitigation strategies across all participants should have minimized differential bias between groups. The survey relies on self-reported data, which may be affected by recall bias, especially regarding occupational exposure, duration of exposure, food consumption and heat stress assessment. Additionally, seasonal variations significantly influence the type and intensity of heat stress and pesticide use. Some individuals engage in multiple occupations as a source of income, leading to potential inaccuracies and overlapping information in their responses. These potential confounders such as recall bias, seasonal variability in exposures (e.g., heat stress, pesticide application) and occupational shifts may affect the accuracy of risk factor assessment. Future studies should incorporate longitudinal designs to minimise these biases. Also, the choice of interval for test–retest reliability can influence results. While our two-week interval aimed to balance various factors, we recognise that some temporal effects may still be present.

The developed questionnaire could be integrated in various healthcare settings to support early detection and risk assessment of CKDu, including 

i. Primary Care, as a screening tool for individuals with non-specific symptoms like fatigue, dehydration, or mild renal abnormalities.
• Speciality Clinics: identifying the need for interventions like hydration, occupational safety recommendations, and dietary modifications.
• Furthermore, community health workers (e.g., ASHA workers, nurses) can administer the tool during field visits, particularly in occupationally-exposed populations.
ii. The tool can be implemented via paper-based forms or integrated into electronic health records (EHRs), with potential for mobile applications to enable real-time assessments.
iii. Insights from questionnaire data can support public health policies by identifying high-risk trends and advocating for targeted interventions in vulnerable communities.

## Supplementary Information

Below is the link to the electronic supplementary material.

Supplementary file1 (PDF 1484 KB)

## Data Availability

The original contributions presented in this study are included in the article/supplementary material, further enquiry can be directed to the corresponding author.
