# Supplementary material for: Development, validation, and reliability of a questionnaire to assess risk-factors of chronic kidney disease of unknown etiology
Source: J Nephrol. 2025 May 12;38(6):1583–94. doi: 10.1007/s40620-025-02297-3 (PMC12378130; doi:10.1007/s40620-025-02297-3)
Supplement: Supplementary file 1 — Supplementary file1 (PDF 1484 KB) [file 40620_2025_2297_MOESM1_ESM.pdf]

STROBE Statement—Checklist of items that should be included in reports of ***cross-sectional studies***

	Item No	Recommendation	Page No
**Title and abstract**	1	(*a*) Indicate the study's design with a commonly used term in the title or the abstract	3
		(*b*) Provide in the abstract an informative and balanced summary of what was done and what was found	3
**Introduction**			
Background/rationale	2	Explain the scientific background and rationale for the investigation being reported	7-9
Objectives	3	State specific objectives, including any prespecified hypotheses	7-9
**Methods**			
Study design	4	Present key elements of study design early in the paper	9
Setting	5	Describe the setting, locations, and relevant dates, including periods of recruitment, exposure, follow-up, and data collection	9-15
Participants	6	(*a*) Give the eligibility criteria, and the sources and methods of selection of participants	13
Variables	7	Clearly define all outcomes, exposures, predictors, potential confounders, and effect modifiers. Give diagnostic criteria, if applicable	9-15
Data sources/ measurement	8*	For each variable of interest, give sources of data and details of methods of assessment (measurement). Describe comparability of assessment methods if there is more than one group	9-15
Bias	9	Describe any efforts to address potential sources of bias	NA
Study size	10	Explain how the study size was arrived at	11
Quantitative variables	11	Explain how quantitative variables were handled in the analyses. If applicable, describe which groupings were chosen and why	8-13
Statistical methods	12	(*a*) Describe all statistical methods, including those used to control for confounding	14
		(*b*) Describe any methods used to examine subgroups and interactions	NA
		(*c*) Explain how missing data were addressed	14
		(*d*) If applicable, describe analytical methods taking account of sampling strategy	14
		(*e*) Describe any sensitivity analyses	NA
**Results**			
Participants	13*	(a) Report numbers of individuals at each stage of study—eg numbers potentially eligible, examined for eligibility, confirmed eligible, included in the study, completing follow-up, and analysed	15-17
		(b) Give reasons for non-participation at each stage	15-17
		(c) Consider use of a flow diagram	9
Descriptive data	14*	(a) Give characteristics of study participants (eg demographic, clinical, social) and information on exposures and potential confounders	16-17
		(b) Indicate number of participants with missing data for each variable of interest	16-17
Outcome data	15*	Report numbers of outcome events or summary measures	NA

Main results	16	(*a*) Give unadjusted estimates and, if applicable, confounder-adjusted estimates and their precision (eg, 95% confidence interval). Make clear which confounders were adjusted for and why they were included	15-17
		(*b*) Report category boundaries when continuous variables were categorized	NA
		(*c*) If relevant, consider translating estimates of relative risk into absolute risk for a meaningful time period	NA
Other analyses	17	Report other analyses done—eg analyses of subgroups and interactions, and sensitivity analyses	NA
**Discussion**			
Key results	18	Summarise key results with reference to study objectives	17-20
Limitations	19	Discuss limitations of the study, taking into account sources of potential bias or imprecision. Discuss both direction and magnitude of any potential bias	18
Interpretation	20	Give a cautious overall interpretation of results considering objectives, limitations, multiplicity of analyses, results from similar studies, and other relevant evidence	17-20
Generalisability	21	Discuss the generalisability (external validity) of the study results	NA
**Other information**			
Funding	22	Give the source of funding and the role of the funders for the present study and, if applicable, for the original study on which the present article is based	22

*Give information separately for exposed and unexposed groups.

Note: An Explanation and Elaboration article discusses each checklist item and gives methodological background and published examples of transparent reporting. The STROBE checklist is best used in conjunction with this article (freely available on the Web sites of PLoS Medicine at http://www.plosmedicine.org/, Annals of Internal Medicine at http://www.annals.org/, and Epidemiology at http://www.epidem.com/). Information on the STROBE Initiative is available at www.strobe-statement.org.

Supplementary file S1: Draft questionnaire before validation

**Personal information**
1. Age
2. Marital status (Married/ unmarried/ widower)
3. Gender
4. Education (literate/ illiterate)
5. Qualification
6. Family income
7. No: of family members
8. Occupation (farmer/ driver/ Coolie/ fisherman)
9. Previous occupation, if any
10. Family history of kidney disease (Yes/ No). If yes, then;
11. Family history other than kidney disease (mother/ father/ sister/ brother). If yes, then;
12. Past medical history, if any
13. Past medication history, if any
14. Social history (Yes/ No). If yes, then; details<br>a. smoking b. snuffs c. alcohol (ethanol) d. alcohol (toddy) e. haans f. paan g. ghutka
**Past health issues (related to kidney)**
1. Do you have any history of kidney problems at birth or in childhood? If yes, then;<br>a. diagnosis b. age of diagnosis c. treatment
2. Have you ever been told or diagnosed with kidney disease? If yes, then;<br>a. diagnosis b. duration since diagnosed c. treatment
3. Did you ever have a history of anaemia or low haemoglobin levels? If yes, then;<br>a. type of anaemia b. duration since diagnosed c. treatment
4. Have you ever suffered any bone and/or joint pain(s)?<br>If yes, then;<br>a. site of pain(s) b. frequency c. treatment
5. Have you ever faced any issues during urination in the past? If yes, then;<br>a. details of the issue b. duration c. treatment
6. Have you ever done any urine test in the past? If yes, then;<br>a. reason for testing b. results c. treatment
7. Did you ever have any urinary infections in the past? If yes, then;<br>a. diagnosis b. age at diagnosis c. treatment
8. Did you ever undergo any ultrasound, Xray, CT, MRI or any other type of radiation? If yes, then;<br>a. reason for exposure b. diagnosis, if any c. treatment
9. Have you ever experienced swelling of feet/legs/eyes/eyelids? If yes, possible reason?
10. When you have an illness, which type of medicine do you generally use?<br>a. allopathic b. ayurvedic c. folk medicine d. homeopathic e. others
11. Are you taking any vitamin supplements without doctor’s advice? If yes, then;<br>a. name of the product b. composition c. duration/frequency of intake d. reason (if known)
12. Has any healthcare professional advised you a salt restricted diet anytime in the past? If yes, then;<br>a. designation of HCP b. reason for advice c. type of salt used
**Agrochemicals**
1. Do you cultivate any vegetables and fruits at your home premises? If yes, then;<br>a. source of watering b. fertilizers/pesticides used if any c. brand d. frequency of spraying

2. Do you use any pesticides for cultivation of crops? If yes, then;<br>a. name of the pesticide used b. frequency of exposure c. total duration of exposure
3. Do you have the habit of using any protectants while handling fertilizers or pesticides? Yes/No
4. Do you store any pesticides at your home after use? If yes, then;<br>a. location of storage b. containers used (if any)
5. Do you use any insecticides to prevent mites and ants at your home? If yes, then;<br>a. brand name b. frequency of spraying c. storage
6. Do you have any pets at home? if yes, then;<br>a. Type of pet b. no: of pet c. anti-mites used, if any d. frequency of use
7. Do you wash your hands with soap after returning from the farms? Yes/No
**Occupation**
1. Working habit of the participants<br>a. No: of days in a week b. No: of hours in a day
2. How often do you hydrate yourself with water or any other liquids in between work?<br>a. frequency of hydration b. quantity c. type of fluids
3. How frequently do you urinate in between work?
**OTC Medications**
1. Do you take any medications for minor ailments yourself? If yes, then;<br>a. name of the drug b. reason c. frequency d. duration
2. Do you use home-remedies for general ailments? If yes, then;<br>a. remedy used b. frequency c. reason
3. Have you taken any medicines for pain directly from medical shops? If yes, then;<br>a. name of the medicine b. frequency c. duration
4. Approximately how much water do you consume for taking medicines?
**Drinking water**
1. What is your source of drinking water? Well water/ hand pump/ tap water/ borewell water/ other
2. Details about well water<br>a. depth of well b. location c. frequency of cleaning d. age of the well
3. Does your well water mix with ground water? Yes/ No
4. Do you use a water purifier for drinking water? If yes, then;<br>a. age of purifier b. frequency of cleaning c. brand
5. Does your residence have any industries nearby? If yes, then;<br>a. type of industry b. waste products dumped from industries to nearby areas (if any)
6. Do you do rattles and poultry farming? If yes, then;<br>a. source of drinking water b. location of shed c. source of food d. source of bathing water
**Heat stress**
1. For how many hours do you get exposed to sunlight in a day?
2. Do you protect yourself from heat and sunlight during work? If yes, then; mode of protection?
**Air pollution**
1. What is the manner of disposal of waste at your home?<br>a. household garbage b. plant waste c. animal waste d. agricultural waste e. other waste (to specify)
2. What is the source of gas for cooking food at your home?<br>a. LPG b. electrical c. traditional d. biofuel e. others
3. If it’s a traditional way of cooking by not involving LPG then what are the components of the fuel?

a. wood b. plant dry parts c. kerosene d. plastic waste e. coal f. cow dung
4. What vessels do you generally use for cooking food?<br>a. steel b. mud c. aluminium d. copper e. non-stick f. iron
5. Do you ignite fire in earthern stoves through a blow pipe? Yes/ No
**Worm infestations**
1. Do you have a regular practice of deworming? If yes, then;<br>a. type of practice b. frequency
2. Do you have any history of worm infestations? Yes/ No
3. What is the general management you follow for worm infestations?<br>a. allopathic b. ayurvedic c. folk medicine d. homeopathic e. others
**Food and nutrition**
1. Are you a vegetarian/ non-vegetarian/ mixed?
2. Which is the most common type of non-vegetarian food you consume?<br>Fish/ Chicken (broiler/ non-broiler) Egg/ Mutton/ Pork/ Others<br>a. Source of meat and fish in your community b. fresh/frozen
3. Do you preserve food at home? If yes, then;<br>a. method of preservation b. type of food stored c. duration of storage d. preservatives used, if any
4. Which vessels/ containers do you generally use for storing food and water for long time?
5. How often do you take soft drinks or soda?<br>a. name of the drink b. brand name c. frequency
6. How often do you consume tea/coffee in between work?<br>a. type b. quantity c. frequency
7. Do you generally consume home prepared food or outside food? If outside food, then specify;<br>a. hotel food b. canteen/mess food c. instant/fast/junk food d. frequency of consumption per week
8. Do you have the habit of skipping meals: If yes, then specify;<br>a. food intake in a day b. skipping pattern

Supplementary IIa: Questionnaire in English

Questionnaire for assessing the risk factors of chronic kidney disease of unknown etiology

I. ***Personal Information***

Subject Code: ___________ Age: ____________

Marital status: (Married/ Unmarried/ Widower)

RMCW Code:____________ Gender: ____________

Education: (Literate/ Illiterate)

Qualification: ___________ Family income: __________

No. of family members: ______

Occupation: (Farmer/ Driver/ Coolie/ Fisherman)

Previous occupation, if any: __________________________

Family history of kidney disease: (Yes/ No)

If Yes, _________________________________

Family history other than kidney disease: (Mother/ Father/ Sister/ Brother)

If Yes, ______________________________________

Past medical history, if any: _____________________

Past medication history, if any: __________________

Social History (Yes/ No)

Smoking	Snuffs (Tobacco)	Alcohol (Ethanol)
No. of cigarettes a day:<br>Duration:<br>Brand used:<br>Others:	Brand used:<br>Frequency:<br>Duration:	Frequency:<br>Duration:<br>Brand used:<br>Quantity per day:

Alcohol (Toddy)	Haans	Paan
Frequency:<br>Quantity per day:<br>Duration:	Frequency:<br>Duration:	Frequency:<br>Duration:

Ghutka
Frequency of chewing:<br>Duration:

II. Past health issues (related to kidney)

1. Do you have any history of kidney problems at birth or in childhood?

If yes, then;

Diagnosis:	Age at diagnosis:	Treatment/duration:

2. Have you ever been told or diagnosed with kidney disease?

If yes, then;

Diagnosis:	Duration since diagnosed:	Treatment:

3. Did you ever had a history of anaemia or low haemoglobin levels?

If yes, then;

Type of anaemia:	Duration since diagnosed:	Treatment:

4. Have you ever suffered any bone and/or joint pain(s)?

If yes, then;

Site of pain(s):	Frequency:	Treatment:

5. Have you ever faced any issues during urination in the past?

If yes, then;

Details of the issue:	Duration:	Treatment:

6. Have you ever done any urine test in the past?

If yes, then;

Reason for testing:	Results:	Treatment:

7. Did you ever have any urinary infections in the past?

If yes, then;

Diagnosis:	Age at diagnosis:	Treatment:

8. Did you ever undergo any ultrasound, X ray, CT, MRI or any other type of radiation?

If yes, then;

Reason for exposure:	Diagnosis, if any:	Treatment:

9. Have you ever experienced swelling of feet/legs/eyes/eyelids?

If yes, possible reason: ___________________________

10. When you have an illness, which type of medicine do you generally use?

Allopathic	Ayurvedic	Folk medicine	Homeopathic	Others

11. Are you taking any vitamin supplements without doctor's advice?

If yes, then;

Name of the product:	Composition:	Duration/frequency of intake:
Reason (if known):		

12. Has any healthcare professional advised you a salt restricted diet anytime in the past?

If yes, then;

Designation of HCP:	Reason for advice:	Type of salt used:

III. Agrochemicals

1. Do you cultivate any vegetables and fruits at your home premises?

If yes, then;

Source of watering:	Fertilizers/pesticides used if any:
Brand:	Frequency of spraying:

2. Do you use any pesticides for cultivation of crops?

If yes, then;

Name of the pesticide used:	Frequency of exposure:
Total duration of exposure:	

3. Do you have the habit of using any protectants while handling fertilizers or pesticides?

Yes/ No

4. Do you store any pesticides at your home after use?

If yes, then;

Location of storage:	Containers used, (if any):

5. Do you use any insecticides to prevent mites and ants at your home?

If yes, then;

Brand name:	Frequency of spraying:	Storage:

6. Do you have any pets at home?

If yes, then;

Type of pet:	No. of pets:
Anti-mites used, if any:	Frequency of use:

7. Do you wash your hands with soap after returning from the farms?

Yes/ No

IV. Occupation

1. Working habit of the participants:

No. of days in a week:	No. of hours in a day:

2. How often do you hydrate yourself with water or any other liquids in between work?

Frequency of hydration:	Quantity:	Type of fluids:

V. OTC Medications

1. Do you take any medications for minor ailments yourself?

If yes, then;

Allopathy/ Ayurveda/ Homeopathy/ Siddha	
Name of drug:	Reason:
Frequency:	Duration:

2. Do you use home-remedies for general ailments?

If yes, then;

Remedy used:	Frequency:	Reason:

3. Have you ever taken any medicines for pain directly from medical shops?

If yes, then;

Indication: Chronic pain to knee or back/ Acute intermittent pain/ menstrual pain/ occasional pain/ body pain / headache/ Others: ___________________________________		
Name of the medicine:	Frequency:	Duration:

4. Approximately how much water do you consume for taking medicines? _______________

VI. Drinking water

1. What is your source of drinking water?

Well water/ Hand pump/ Tap water/ Borewell water/ Other:__________

2. Details about well water:

Depth of well:	Location:	Frequency of cleaning:
Age of the well:		

3. Does your well water mix with ground water? Yes/ No

4. Do you use a water purifier for drinking water?

If yes, then;

Age of purifier:	Frequency of cleaning:	Brand:

5. Does your residence have any industries nearby?

If yes, then

Type of industry:
Waste product(s) dumped from industries to nearby areas (if any):

6. Do you do rattles and poultry farming?

If yes, then;

Source of drinking water:	Location of shed:	Source of food:
Source of bathing water:		

VII. Heat stress

1. For how many hours do you get exposed to sunlight in a day? ________________________

2. Do you protect yourself from heat and sunlight during work?

If yes, then; Mode of protection: _________________________

VIII. Air Pollution

1. What is the manner of disposal of waste at your home?

Burn/ disposing using plastics into river or lakes/ Biogas plants/ Municipality or panchayat services/ Others
Household garbage:
Plant waste:
Animal waste:
Agricultural waste:
Other waste (to specify):

2. What is the source of gas for cooking food at your home?

LPG/ Electrical/ Traditional/ Biofuel/Others

3. If it's a traditional way of cooking by not involving LPG, then what are the components of the fuel?

Wood/ Plant dry parts/ Kerosene/ Plastic waste/ Coal/ Cow dung

4. What vessels do you generally use for cooking food?

Steel/ mud/ aluminium/ copper/ non-stick/ iron

IX. Worm infestations

1. Do you have a regular practice of deworming?

If yes, then;

Type of practice:	Frequency:

2. Do you have any history of worm infestation? Yes/ No

3. What is the general management you follow for worm infestations?

Allopathic	Ayurvedic	Folk medicine	Homeopathic	Others

X. Food and Nutrition

1. Are you a vegetarian/ non-vegetarian/ mixed? _________________________

2. Which is the most common type of non-vegetarian food you consume?

Fish/ chicken (broiler/non-broiler)/ Egg/ Mutton/ Pork/Others	
Source of meat and fish in your community:	Fresh/frozen

3. Do you preserve food at home?

If yes, then;

Method of preservation:	Type of food stored:
Duration of storage:	Preservatives used, if any:

4. Which vessels/containers do you generally use for storing food and water for long time?

____

5. How often do you take soft drinks or soda?

Name of the drink:	Brand name:
Frequency:	

6. How often do you consume tea/coffee in between work?

Type:	Quantity:
Frequency:	

7. Do you generally consume home prepared food or outside food?

If outside food, then specify

Hotel food:	Canteen/mess food:	Instant/fast/junk food:
Frequency of consumption per week:		

8. Do you have a habit of skipping meals?

If yes, then specify

Food intake in a day	Skipping pattern		
	Never	Occasionally	Frequently
Breakfast			
Lunch			
Dinner			

ಅಜ್ಞಾತ ರೋಗಶಾಸ್ತ್ರದ ದೀರ್ಘಕಾಲದ ಮೂತ್ರಪಿಂಡ ಕಾಯಿಲೆಯ ಅಪಾಯ ಅಂಶಗಳನ್ನು ನಿರ್ಣಯಿಸುವ ಪ್ರಶ್ನಾವಳಿ

I. ವೈಯಕ್ತಿಕ ಮಾಹಿತಿ

ವಿಷಯ ಕೋಡ್: ___________ ವಯಸ್ಸು: ____________ ವೈವಾಹಿಕ ಸ್ಥಿತಿ: (ವಿವಾಹಿತ/ ಅವಿವಾಹಿತ/ ವಿಧುರ)

RMCW ಕೋಡ್:____________ ಲಿಂಗ: ____________ ಶಿಕ್ಷಣ: (ಅಕ್ಷರಸ್ಥ/ಅನಕ್ಷರಸ್ಥ)

ವಿದ್ಯಾರ್ಹತೆ: ___________ ಕುಟುಂಬದ ಆದಾಯ: ________ಕುಟುಂಬ ಸದಸ್ಯರ ಸಂಖ್ಯೆ: ______

ಉದ್ಯೋಗ: (ರೈತ/ಚಾಲಕ/ಕೂಲಿ/ಮೀನುಗಾರ/ಇತರೆ) ಹಿಂದಿನ ಉದ್ಯೋಗ, ಯಾವುದಾದರೂ ಇದ್ದರೆ ನಿರ್ದಿಷ್ಟಪಡಿಸಿ ___________________________

ಮೂತ್ರಪಿಂಡ ಕಾಯಿಲೆಯ ಕುಟುಂಬದ ಇತಿಹಾಸ: (ಹೌದು/ಇಲ್ಲ)

ಹೌದು ಎಂದಾದರೆ, _________________________________

ಮೂತ್ರಪಿಂಡ ಕಾಯಿಲೆ ಹೊರತುಪಡಿಸಿ ಕುಟುಂಬದ ಇತಿಹಾಸ: (ತಾಯಿ/ತಂದೆ/ಸಹೋದರಿ/ ಸಹೋದರ)

ಹೌದು ಎಂದಾದರೆ, _______________________________________

ಹಿಂದಿನ ವೈದ್ಯಕೀಯ ಇತಿಹಾಸ, ಯಾವುದಾದರೂ ಇದ್ದರೆ: _____________________

ಹಿಂದಿನ ಔಷಧಿಯ ಇತಿಹಾಸ, ಯಾವುದಾದರೂ ಇದ್ದರೆ: __________________

ಸಾಮಾಜಿಕ ಇತಿಹಾಸ (ಹೌದು/ಇಲ್ಲ)

ಧೂಮಪಾನ

ಒಂದು ದಿನಕ್ಕೆ ಬಳಸುವ ಸಿಗರೇಟ್ ಗಳ ಸಂಖ್ಯೆ:
ಅವಧಿ:
ಉಪಯೋಗಿಸುವ ಗುರುತು:
ಇತರೆ:

ನಶ್ಯ (ತಂಬಾಕು)

ಉಪಯೋಗಿಸುವ ಗುರುತು:
ಆವರ್ತನೆ
ಅವಧಿ:

ಮದ್ಯ (ಎಥೆನಾಲ್)

ಆವರ್ತನೆ
ಅವಧಿ:
ಉಪಯೋಗಿಸುವ ಗುರುತು:
ಒಂದು ದಿನಕ್ಕೆ ಬಳಸುವ ಅಳತೆ:

ಮದ್ಯ (ಟಾಡಿ)

ಆವರ್ತನೆ
ಒಂದು ದಿನಕ್ಕೆ ಬಳಸುವ ಅಳತೆ:
ಅವಧಿ:

ಹಾನ್ಸ್

ಆವರ್ತನೆ:
ಅವಧಿ:

ಪಾನ್

ಆವರ್ತನೆ:
ಅವಧಿ:

ಗುಟ್ಕಾ

ಜಗಿಯುವ ಆವರ್ತನೆ:
ಅವಧಿ:

II. ಹಿಂದಿನ ಆರೋಗ್ಯ ಸಮಸ್ಯೆಗಳು (ಮೂತ್ರಪಿಂಡಕ್ಕೆ ಸಂಬಂಧಿಸಿದಂತೆ)

1. ನೀವು ಹುಟ್ಟುವಾಗ ಅಥವಾ ಬಾಲ್ಯದಲ್ಲಿ ಮೂತ್ರಪಿಂಡದ ಸಮಸ್ಯೆಗಳ ಇತಿಹಾಸವನ್ನು ಹೊಂದಿದ್ದೀರಾ?

ಹೌದು ಎಂದಾದರೆ;

ರೋಗನಿರ್ಣಯ	ರೋಗನಿರ್ಣಯಕ್ಕೊಳಗಾದ ವಯಸ್ಸು	ಚಿಕಿತ್ಸೆ/ಅವಧಿ

2. ನಿಮಗೆ ಎಂದಾದರೂ ಕಿಡ್ನಿ ಕಾಯಿಲೆ ಇತ್ತೆ ಅಥವಾ ರೋಗನಿರ್ಣಯ ಮಾಡಲ್ಪಟ್ಟಿದ್ದೀರಾ?

ಹೌದು ಎಂದಾದರೆ;

ರೋಗನಿರ್ಣಯ	ರೋಗನಿರ್ಣಯಿಸಲ್ಪಟ್ಟ ನಂತರದ ಅವಧಿ	ಚಿಕಿತ್ಸೆ

3. ನೀವು ಎಂದಾದರೂ ರಕ್ತಹೀನತೆಯ ಇತಿಹಾಸವನ್ನು ಹೊಂದಿದ್ದೀರಾ ಅಥವಾ ಕಡಿಮೆ ಹಿಮೋಗ್ಲೋಬಿನ್ ಮಟ್ಟವನ್ನು ಹೊಂದಿದ್ದೀರಾ?

ಹೌದು ಎಂದಾದರೆ;

ರಕ್ತಹೀನತೆಯ ವಿಧ	ರೋಗನಿರ್ಣಯಿಸಲ್ಪಟ್ಟ ನಂತರದ ಅವಧಿ	ಚಿಕಿತ್ಸೆ

4. ನೀವು ಎಂದಾದರೂ ಯಾವುದೇ ಮೂಳೆ ಮತ್ತು/ಅಥವಾ ಕೀಲು ನೋವು(ಗಳು) ಅನುಭವಿಸಿದ್ದೀರಾ?

ಹೌದು ಎಂದಾದರೆ;

ನೋವು (ಗಳು) ಇರುವ ಜಾಗ	ಆವರ್ತನೆ	ಚಿಕಿತ್ಸೆ

5. ಹಿಂದೆ ಎಂದಾದರೂ ಮೂತ್ರ ವಿಸರ್ಜನೆಯ ಸಮಯದಲ್ಲಿ ನೀವು ಯಾವುದೇ ಸಮಸ್ಯೆಗಳನ್ನು ಎದುರಿಸಿದ್ದೀರಾ?

ಹೌದು ಎಂದಾದರೆ;

ಪ್ರಕರಣದ ಬಗ್ಗೆ ವಿವರಣೆ	ಅವಧಿ	ಚಿಕಿತ್ಸೆ

6. ಕಳೆದ 1 ವರ್ಷದಲ್ಲಿ ನೀವು ಎಂದಾದರೂ ಮೂತ್ರ ಪರೀಕ್ಷೆಯನ್ನು ಮಾಡಿದ್ದೀರಾ?

ಹೌದು ಎಂದಾದರೆ;

ಪರೀಕ್ಷಿಸಲು ಕಾರಣಗಳು	ಫಲಿತಾಂಶಗಳು	ಚಿಕಿತ್ಸೆ

7. ಕಳೆದ 1 ವರ್ಷದಲ್ಲಿ ನೀವು ಎಂದಾದರೂ ಮೂತ್ರದ ಸೋಂಕನ್ನು ಹೊಂದಿದ್ದೀರಾ?

ಹೌದು ಎಂದಾದರೆ;

ರೋಗನಿರ್ಣಯ	ರೋಗನಿರ್ಣಯಕ್ಕೊಳಗಾದ ವಯಸ್ಸು	ಚಿಕಿತ್ಸೆ

8. ನೀವು ಹಿಂದೆ ಎಂದಾದರೂ ಮೂತ್ರಪಿಂಡದ ಕಲನಶಾಸ್ತ್ರದ ಕಾಯಿಲೆಯನ್ನು ಹೊಂದಿದ್ದೀರಾ?

ಹೌದು ಎಂದಾದರೆ;

ರೋಗನಿರ್ಣಯ	ರೋಗನಿರ್ಣಯಕ್ಕೊಳಗಾದ ವಯಸ್ಸು	ಚಿಕಿತ್ಸೆ

9. ನೀವು ಎಂದಾದರೂ ಯಾವುದೇ ಅಲ್ಟ್ರಾಸೌಂಡ್, ಎಕ್ಸ್ ರೇ, CT, MRI ಅಥವಾ ಯಾವುದೇ ರೀತಿಯ ವಿಕಿರಣಕ್ಕೆ ಒಳಗಾಗಿದ್ದೀರಾ?

ಹೌದು ಎಂದಾದರೆ;

ಒಡ್ಡುವಿಕೆಗೆ ಕಾರಣ	ರೋಗನಿರ್ಣಯ ಯಾವುದಾದರು ಇದ್ದಲ್ಲಿ	ಚಿಕಿತ್ಸೆ
ಪರೀಕ್ಷೆಯ ಇಸವಿ		

10. ನೀವು ಎಂದಾದರೂ ಪಾದಗಳು/ಕಾಲುಗಳು/ಕಣ್ಣುಗಳು/ಕಣ್ಣುರೆಪ್ಪೆಗಳ ಊತವನ್ನು ಅನುಭವಿಸಿದ್ದೀರಾ?

ಹೌದು ಎಂದಾದರೆ, ದಯವಿಟ್ಟು ವಿವರಣೆಗಳನ್ನು ನೀಡಿ: ______________________________

11. ನೀವು ಅನಾರೋಗ್ಯವನ್ನು ಹೊಂದಿರುವಾಗ, ನೀವು ಸಾಮಾನ್ಯವಾಗಿ ಯಾವ ರೀತಿಯ ಔಷಧವನ್ನು ಬಳಸುತ್ತೀರಿ?

ಅಲೋಪತಿ	ಆಯುರ್ವೇದ	ಹೋಮಿಯೋಪತಿ	ಹಳ್ಳಿ ಔಷಧಗಳು	ಇತರೆ

12. ವೈದ್ಯರ ಸಲಹೆಯಿಲ್ಲದೆ ನೀವು ಯಾವುದಾದರು ವಿಟಮಿನ್ ಪೂರಕಗಳನ್ನು ತೆಗೆದುಕೊಳ್ಳುತ್ತೀರಾ?

ಹೌದು ಎಂದಾದರೆ;

ಉತ್ಪನ್ನದ ಹೆಸರು	ಸಂಯೋಜನೆ	ತೆಗೆದುಕೊಳ್ಳುವ ಅವಧಿ/ಆವರ್ತನೆ	ಕಾರಣ (ಗೊತ್ತಿದ್ದಲ್ಲಿ)

13. ಯಾವುದೇ ಆರೋಗ್ಯ ವೃತ್ತಿಪರರು ಈ ಹಿಂದೆ ಯಾವುದೇ ಸಮಯದಲ್ಲಿ ಉಪ್ಪು ನಿರ್ಬಂಧಿತ ಆಹಾರವನ್ನು ನಿಮಗೆ ಸಲಹೆ ನೀಡಿದ್ದಾರೆಯೇ?

ಹೌದು ಎಂದಾದರೆ;

ಆರೋಗ್ಯ ವೃತ್ತಿಪರರ(HCP) ಪದನಾಮ	ಸಲಹೆಗೆ ಕಾರಣಗಳು	ಉಪಯೋಗಿಸಿದ ಉಪ್ಪಿನ ವಿಧ

14. ಯಾವುದೇ ಆರೋಗ್ಯ ವೃತ್ತಿಪರರು ನಿಮಗೆ ಈ ಹಿಂದೆ ನಿರ್ಬಂಧಿತ ದ್ರವ/ನೀರಿನ ಸೇವನೆಯನ್ನು ಸಲಹೆ ಮಾಡಿದ್ದಾರೆಯೇ?

ಹೌದಾದರೆ, ಆಗ

ಆರೋಗ್ಯ ವೃತ್ತಿಪರರ(HCP) ಪದನಾಮ	ಸಲಹೆಗೆ ಕಾರಣಗಳು	ಪ್ರಸ್ತುತ ತೆಗೆದುಕೊಳ್ಳುತ್ತಿರುವ ಪ್ರಮಾಣ

III. ಕೃಷಿ ರಾಸಾಯನಿಕಗಳು

1. ನಿಮ್ಮ ಮನೆಯ ಆವರಣದಲ್ಲಿ ನೀವು ಯಾವುದೇ ತರಕಾರಿಗಳು ಮತ್ತು ಹಣ್ಣುಗಳನ್ನು ಬೆಳೆಸುತ್ತೀರಾ?

ಹೌದು ಎಂದಾದರೆ;

ನೀರಿನ ಮೂಲ	ಯಾವುದೇ ಗೊಬ್ಬರ/ರಾಸಾಯನಿಕಗಳನ್ನು ಬಳಸುತ್ತಿದ್ದಲ್ಲಿ
ಗುರುತು	ಸಿಂಪಡಿಸುವ ಆವರ್ತನೆ

2. ನೀವು ಬೆಳೆಗಳ ಕೃಷಿಗೆ ಯಾವುದೇ ಕೀಟನಾಶಕಗಳನ್ನು ಬಳಸುತ್ತೀರಾ?

ಹೌದು ಎಂದಾದರೆ;

ಉಪಯೋಗಿಸಿದ ಕೀಟನಾಶಕದ ಹೆಸರು	ಒಡ್ಡುವಿಕೆಯ ಆವರ್ತನೆ
ಒಡ್ಡುವಿಕೆಯ ಒಟ್ಟು ಅವಧಿ	

3. ರಸಗೊಬ್ಬರಗಳು ಅಥವಾ ಕೀಟನಾಶಕಗಳನ್ನು ನಿರ್ವಹಿಸುವಾಗ ನೀವು ಯಾವುದೇ ರಕ್ಷಕಗಳನ್ನು ಬಳಸುವ ಅಭ್ಯಾಸವನ್ನು ಹೊಂದಿದ್ದೀರಾ?

ಹೌದು / ಅಲ್ಲ

4. ಬಳಸಿದ ನಂತರ ನೀವು ನಿಮ್ಮ ಮನೆಯಲ್ಲಿ ಯಾವುದೇ ಕೀಟನಾಶಕಗಳನ್ನು ಸಂಗ್ರಹಿಸುತ್ತೀರಾ?

ಹೌದು ಎಂದಾದರೆ;

ಸಂಗ್ರಹಣೆಯ ಜಾಗ	ಬಳಸುವ ಪಾತ್ರೆಗಳು (ಯಾವುದಾದರು ಇದ್ದಲ್ಲಿ)	ತೆರೆದಿಟ್ಟ/ಮುಚ್ಚಿದ ಪಾತ್ರೆಗಳು

5. ನಿಮ್ಮ ಮನೆಯಲ್ಲಿ ಸೊಳ್ಳೆಗಳು, ಜಿರಳೆಗಳು, ಹುಳಗಳು ಮತ್ತು ಇರುವೆಗಳನ್ನು ತಡೆಗಟ್ಟಲು ನೀವು ಯಾವುದೇ ಕೀಟನಾಶಕ ನಿವಾರಕ ಸಿಂಪಡಿಕೆಗಳು / ಸುರುಳಿಗಳನ್ನು ಬಳಸುತ್ತೀರಾ?

ಹೌದು ಎಂದಾದರೆ;

ಗುರುತಿನ ಹೆಸರು	ಸಿಂಪಡಿಕೆಯ ಆವರ್ತನೆ	ಸಂಗ್ರಹಣೆ

6. ನೀವು ಮನೆಯಲ್ಲಿ ಯಾವುದೇ ಸಾಕುಪ್ರಾಣಿಗಳನ್ನು ಹೊಂದಿದ್ದೀರಾ?

ಹೌದು ಎಂದಾದರೆ

ಸಾಕುಪ್ರಾಣಿಯ ವಿಧ	ಸಾಕುಪ್ರಾಣಿಯ ಸಂಖ್ಯೆ
ಉಪಯೋಗಿಸುವ ಕೀಟನಾಶಕಗಳು	ಉಪಯೋಗಿಸುವಿಕೆಯ ಆವರ್ತನೆ

7. ಹೊಲಗಳಿಂದ ಹಿಂತಿರುಗಿದ ನಂತರ ನೀವು ನಿಮ್ಮ ಕೈಗಳನ್ನು ಸಾಬೂನಿನಿಂದ ತೊಳೆಯುತ್ತೀರಾ?

ಹೌದು / ಅಲ್ಲ

IV. ಉದ್ಯೋಗ

1. ಭಾಗವಹಿಸುವವರ ಕೆಲಸದ ಅಭ್ಯಾಸ:

ಒಂದು ವಾರದಲ್ಲಿ ಕೆಲಸ ಮಾಡುವ ದಿನಗಳ ಸಂಖ್ಯೆ	ಒಂದು ದಿನಕ್ಕೆ ಕೆಲಸ ಮಾಡುವ ಗಂಟೆಗಳು
ಕೆಲಸದ ವಿಧ<br>(ಕುಳಿತುಕೊಳ್ಳುವುದು, ತುಂಬಾ ಸಮಯದ ವರೆಗೆ ನಿಲ್ಲುವುದು, ತುಂಬಾ ಸಕ್ರಿಯ, ದೈಹಿಕ ಶ್ರಮವಿಲ್ಲದ)	

2. ಕೆಲಸದ ನಡುವೆ ನೀವು ಎಷ್ಟು ಬಾರಿ ನೀರು ಅಥವಾ ಇನ್ನಾವುದೇ ದ್ರವದಿಂದ ನಿಮ್ಮನ್ನು ತೇವಾಂಶದಿಂದ ಇರುವಂತೆ ಮಾಡಿಕೊಳ್ಳುತ್ತೀರಿ?

ಕುಡಿಯುವ ಆವರ್ತನೆ	ಪ್ರಮಾಣ	ದ್ರವದ ವಿಧ

3. ನೀವು ದಿನಕ್ಕೆ ಎಷ್ಟು ಬಾರಿ ಮೂತ್ರ ವಿಸರ್ಜಿಸುತ್ತೀರಿ?

V. OTC ಔಷಧಿಗಳು

1. ಸಣ್ಣಪುಟ್ಟ ಕಾಯಿಲೆಗಳಿಗೆ ನೀವೇನಾದರೂ ಔಷಧಿಗಳನ್ನು ತೆಗೆದುಕೊಳ್ಳುತ್ತೀರಾ?

ಹೌದು ಎಂದಾದರೆ;

ಅಲೋಪಥಿ/ ಆಯುರ್ವೇದ/ ಹೋಮಿಯೋಪಥಿ / ಸಿದ್ಧ ಔಷಧಗಳು	
ಔಷಧಿಯ ಹೆಸರು	ಕಾರಣ
ಆವರ್ತನೆ	ಅವಧಿ

2. ನೀವು ಸಾಮಾನ್ಯ ಕಾಯಿಲೆಗಳಿಗೆ ಮನೆ-ಮದ್ದುಗಳನ್ನು ಬಳಸುತ್ತೀರಾ?

ಹೌದು ಎಂದಾದರೆ;

ಮನೆ ಮದ್ದಿನ ಹೆಸರು	ಆವರ್ತನೆ	ಕಾರಣ

3. ನೀವು ಎಂದಾದರೂ ನೋವಿಗೆ ಯಾವುದೇ ಔಷಧಿಗಳನ್ನು ನೇರವಾಗಿ ಔಷಧಿ ಅಂಗಡಿಗಳಿಂದ ತೆಗೆದುಕೊಂಡಿದ್ದೀರಾ?

ಹೌದು ಎಂದಾದರೆ

ಸೂಚನೆ: ಮೊಣಕಾಲು ಅಥವಾ ಬೆನ್ನಿಗೆ ದೀರ್ಘಕಾಲದ ನೋವು/ ತೀವ್ರ ಮರುಕಳಿಸುವ ನೋವು/ ಮುಟ್ಟಿನ ನೋವು/ ಸಾಂದರ್ಭಿಕ ನೋವು/ ದೇಹದ ನೋವು/ ತಲೆನೋವು/ ಇತರೆ: ________________________________		
ಔಷಧದ ಹೆಸರು:	ಆವರ್ತನೆ:	ಅವಧಿ

4. ಔಷಧಿಗಳನ್ನು ತೆಗೆದುಕೊಳ್ಳಲು ನೀವು ಅಂದಾಜು ಎಷ್ಟು ನೀರು ಸೇವಿಸುತ್ತೀರಿ? ______________

VI ಕುಡಿಯುವ ನೀರು

1. ನಿಮ್ಮ ಕುಡಿಯುವ ನೀರಿನ ಮೂಲ ಯಾವುದು?

ಬಾವಿ ನೀರು /ಕೈ ಪಂಪ್ /ನಳ್ಳಿ ನೀರು /ಬೋರ್ವೆಲ್ ನೀರು /ಇತರೆ :___________

2. ಬಾವಿ ನೀರಿನ ಬಗ್ಗೆ ವಿವರಗಳು:

ಬಾವಿಯ ಆಳ:	ಸ್ಥಳ:	ಶುಚಿಗೊಳಿಸುವ ಆವರ್ತನ:
ಬಾವಿಯ ವಯಸ್ಸು:		

3. ನಿಮ್ಮ ಬಾವಿ ನೀರು ಅಂತರ್ಜಲದೊಂದಿಗೆ ಬೆರೆತಿದೆಯೇ? ಹೌದು /ಅಲ್ಲ

4. ನೀವು ಕುಡಿಯುವ ನೀರಿಗೆ ನೀರು ಶುದ್ಧೀಕರಣದ ಸಾಧನವನ್ನು ಬಳಸುತ್ತೀರಾ?

ಹೌದು ಎಂದಾದರೆ;

ಶುದ್ಧೀಕರಣದ ವಯಸ್ಸು:	ಶುಚಿಗೊಳಿಸುವ ಆವರ್ತನ:	ಗುರುತು:

5. ಶುಧ್ಧೀಕರಣದ ಸಾಧನವು ಲಭ್ಯವಿಲ್ಲದಿದ್ದಲ್ಲಿ, ನೀವು ನೀರನ್ನು ಹೇಗೆ ಸೇವಿಸುತ್ತೀರಿ?

ಕುದಿಸಿದ ನೀರು / ತಂಪಾದ ನೀರು

6. ನಿಮ್ಮ ನಿವಾಸವು ಸಮೀಪದಲ್ಲಿ ಯಾವುದೇ ಕೈಗಾರಿಕೆಗಳನ್ನು ಹೊಂದಿದೆಯೇ?

ಹೌದಾದರೆ, ಆಗ

ಕೈಗಾರಿಕೆಯ ಪ್ರಕಾರ:
ಕೈಗಾರಿಕೆಗಳಿಂದ ಹತ್ತಿರದ ಪ್ರದೇಶಗಳಿಗೆ (ಯಾವುದಾದರೂ ಇದ್ದರೆ) ತ್ಯಾಜ್ಯ ಉತ್ಪನ್ನ(ಗಳು):

7. ನೀವು ಯಾರ್ಟಲ್ಸ್ ಮತ್ತು ಕೋಳಿ ಸಾಕಣೆ ಮಾಡುತ್ತೀರಾ?

ಹೌದು ಎಂದಾದರೆ;

ಕುಡಿಯುವ ನೀರಿನ ಮೂಲ: ಶೆಡ್‌ನ ಸ್ಥಳ: ಆಹಾರದ ಮೂಲ:
ಸ್ನಾನದ ನೀರಿನ ಮೂಲ

VII. ಶಾಖದ ಒತ್ತಡ

1. ಒಂದು ದಿನದಲ್ಲಿ ನೀವು ಎಷ್ಟು ಗಂಟೆಗಳ ಕಾಲ ಸೂರ್ಯನ ಬೆಳಕಿಗೆ ಒಡ್ಡಿಕೊಳ್ಳುತ್ತೀರಿ?

____

2. ಕೆಲಸದ ಸಮಯದಲ್ಲಿ ನೀವು ಶಾಖ ಮತ್ತು ಸೂರ್ಯನ ಬೆಳಕಿನಿಂದ ನಿಮ್ಮನ್ನು ರಕ್ಷಿಸಿಕೊಳ್ಳುತ್ತೀರಾ?

ಹೌದು ಎಂದಾದರೆ; ರಕ್ಷಣೆಯ ವಿಧಾನ: ______________________

VIII. ವಾಯು ಮಾಲಿನ್ಯ

1. ನಿಮ್ಮ ಮನೆಯಲ್ಲಿ ತ್ಯಾಜ್ಯವನ್ನು ವಿಲೇವಾರಿ ಮಾಡುವ ವಿಧಾನ ಯಾವುದು?

ನದಿ ಅಥವಾ ಸರೋವರಗಳಿಗೆ / ಜೈವಿಕ ಅನಿಲ ಸ್ಥಾವರಗಳಿಗೆ /
ಪುರಸಭೆ ಅಥವಾ ಪಂಚಾಯತ್ ಸೇವೆಗಳಿಗೆ / ಇತರರಿಗೆ ಪ್ಲಾಸ್ಟಿಕ್ ಬಳಸಿ ಸುಡುವುದು / ವಿಲೇವಾರಿ ಮಾಡುವುದು

ಮನೆಯ ಕಸ:

ಸಸ್ಯ ತ್ಯಾಜ್ಯ:

ಪ್ರಾಣಿ ತ್ಯಾಜ್ಯ:

ಕೃಷಿ ತ್ಯಾಜ್ಯ:

ಇತರ ತ್ಯಾಜ್ಯ (ನಿರ್ದಿಷ್ಟಗೊಳಿಸಲು):

2. ನಿಮ್ಮ ಮನೆಯಲ್ಲಿ ಆಹಾರಕ್ಕಾಗಿ ಬಳಸುವ ಅಡುಗೆ ಅನಿಲದ ಮೂಲ ಯಾವುದು?

LPG/ ಎಲೆಕ್ಟ್ರಿಕಲ್/ ಸಾಂಪ್ರದಾಯಿಕ/ ಜೈವಿಕ ಇಂಧನ/ಇತರ

3. ಇದು LPG ಯನ್ನು ಒಳಗೊಂಡಿಲ್ಲದ ಸಾಂಪ್ರದಾಯಿಕ ಅಡುಗೆ ವಿಧಾನವಾಗಿದ್ದರೆ, ಇಂಧನದ ಅಂಶಗಳು ಯಾವುವು?

ಮರ/ ಸಸ್ಯ ಒಣ ಭಾಗಗಳು/ ಸೀಮೆ ಎಣ್ಣೆ/ ಪ್ಲಾಸ್ಟಿಕ್ ತ್ಯಾಜ್ಯ/ ಕಲ್ಲಿದ್ದಲು/ ಇತರೆ

4. ಆಹಾರದ ಶೇಖರಣೆಗಾಗಿ ಮತ್ತು ಅಡುಗೆ ಮಾಡಲು ನೀವು ಸಾಮಾನ್ಯವಾಗಿ ಯಾವ ಪಾತ್ರೆಗಳನ್ನು ಬಳಸುತ್ತೀರಿ?

ಸ್ಟೀಲ್/ ಮಣ್ಣು/ ಅಲ್ಯೂಮಿನಿಯಂ/ ತಾಮ್ರ/ ನಾನ್ ಸ್ಟಿಕ್/ ಕಬ್ಬಿಣ/ ಇತರೆ

IX. ಹುಳುಗಳ ಮುತ್ತಿಕೊಳ್ಳುವಿಕೆ

1. ನೀವು ಜಂತುಹುಳು ನಿವಾರಣೆಯ ನಿಯಮಿತ ಅಭ್ಯಾಸವನ್ನು ಹೊಂದಿದ್ದೀರಾ?

ಹೌದು ಎಂದಾದರೆ;

ಅಭ್ಯಾಸದ ವಿಧ	ಆವರ್ತನೆ

2. ಕಳೆದ 1 ವರ್ಷದಲ್ಲಿ ನೀವು ಯಾವುದೇ ಹುಳುಗಳ ಬಾಧೆಯ ಇತಿಹಾಸವನ್ನು ಹೊಂದಿದ್ದೀರಾ? ಹೌದು ಅಲ್ಲ

3. ಹುಳುಗಳ ಮುತ್ತಿಕೊಳ್ಳುವಿಕೆಗೆ ನೀವು ಅನುಸರಿಸುವ ಸಾಮಾನ್ಯ ನಿರ್ವಹಣೆ ಏನು?

ಅಲೋಪಥಿ	ಆಯುರ್ವೇದ	ಜನಪದ ಔಷಧಗಳು	ಹೋಮಿಯೋಪಥಿ	ಇತರೆ

X. ಆಹಾರ ಮತ್ತು ಪೋಷಣೆ

1. ನೀವು ಸಸ್ಯಾಹಾರಿ/ ಮಾಂಸಾಹಾರಿ/ ಮಿಶ್ರಾಹಾರಿಯೇ? ____________________________

2. ನೀವು ಸೇವಿಸುವ ಅತ್ಯಂತ ಸಾಮಾನ್ಯವಾದ ಮಾಂಸಾಹಾರಿ ಆಹಾರ ಯಾವುದು?

ಮೀನು/ಕೋಳಿ (ಬ್ರಾಯ್ಲರ್/ಬ್ರಾಯ್ಲರ್ ಅಲ್ಲದ)/ ಮೊಟ್ಟೆ/ಮಟನ್/ಹಂದಿ/ಇತರ	
ನಿಮ್ಮ ಸಮುದಾಯದಲ್ಲಿ ಮಾಂಸ ಮತ್ತು ಮೀನಿನ ಮೂಲ:	ತಾಜಾ/ಹೆಪ್ಪುಗಟ್ಟಿದ

3. ನೀವು ಮನೆಯಲ್ಲಿ ಆಹಾರವನ್ನು ಸಂರಕ್ಷಿಸುತ್ತೀರಾ?

ಹೌದು ಎಂದಾದರೆ;

ಸಂರಕ್ಷಿಸುವ ವಿಧಾನ:	ಸಂಗ್ರಹಿಸಿದ ಆಹಾರದ ಪ್ರಕಾರ:
ಶೇಖರಣೆಯ ಅವಧಿ:	ಸಂರಕ್ಷಕಗಳನ್ನು ಬಳಸಲಾಗುತ್ತದೆ, ಯಾವುದಾದರೂ ಇದ್ದರೆ:

4. ದೀರ್ಘಾವಧಿಯ ವರೆಗೆ ಆಹಾರ ಮತ್ತು ನೀರನ್ನು ಸಂಗ್ರಹಿಸಲು ನೀವು ಸಾಮಾನ್ಯವಾಗಿ ಯಾವ ಪಾತ್ರೆಗಳು/ಧಾರಕಗಳನ್ನು ಬಳಸುತ್ತೀರಿ?

____

5. ನೀವು ಎಷ್ಟು ಬಾರಿ ತಂಪು ಪಾನೀಯಗಳು ಅಥವಾ ಸೋಡಾವನ್ನು ತೆಗೆದುಕೊಳ್ಳುತ್ತೀರಿ?

ಪಾನೀಯದ ಹೆಸರು	ಗುರುತಿನ ಹೆಸರು
ಆವರ್ತನೆ	

6. ಕೆಲಸದ ನಡುವೆ ನೀವು ಎಷ್ಟು ಬಾರಿ ಟೀ/ಕಾಫಿ ಸೇವಿಸುತ್ತೀರಿ?

ವಿಧ	ಪ್ರಮಾಣ
ಆವರ್ತನೆ	

7. ನೀವು ಸಾಮಾನ್ಯವಾಗಿ ಮನೆಯಲ್ಲಿ ತಯಾರಿಸಿದ ಆಹಾರವನ್ನು ಇಷ್ಟಪಡುತ್ತೀರಾ ಅಥವಾ ಹೊರಗಿನ ಆಹಾರವನ್ನು ಇಷ್ಟಪಡುತ್ತೀರಾ?

ಹೊರಗಿನ ಆಹಾರವಾಗಿದ್ದರೆ, ಸೂಚಿಸಿ

ಹೋಟೆಲ್ ಆಹಾರ:	ಕ್ಯಾಂಟೀನ್/ಮೆಸ್ ಆಹಾರ:	ತ್ವರಿತ/ಫಾಸ್ಟ್/ಜಂಕ್ ಫುಡ್:
ವಾರಕ್ಕೆ ಬಳಕೆಯ ಆವರ್ತನೆ:		

8. ನಿಮಗೆ ಊಟ ಬಿಡುವ ಅಭ್ಯಾಸವಿದೆಯೇ?

ಹೌದು ಎಂದಾದರೆ, ನಿರ್ದಿಷ್ಟಪಡಿಸಿ

ಒಂದು ದಿನದಲ್ಲಿ ಬಳಸುವ ಆಹಾರ	ಊಟ ಬಿಡುವ ಪ್ರಕಾರ		
	ಇಲ್ಲವೇ ಇಲ್ಲ	ಸಾಂದರ್ಭಿಕವಾಗಿ	ಆಗಾಗ
ಬೆಳಗ್ಗಿನ ಉಪಹಾರ			
ಮದ್ಯಾಹ್ನದ ಊಟ			
ರಾತ್ರಿಯ ಊಟ			

Supplementary table SIII: I-CVI content validity

a. **Accuracy**

Question number	Item content validity
Personal information	
1	1
2	0.6
3	1
4	1
5	1
6	1
7	1
8	1
9	0.8
10	1
11	1
12	1
13	1
14	1
Past health issues	
1	1
2	1
3	1
4	0.8
5	1
6	1
7	1
8	1
9	1
10	0.8
11	1
12	1
Agrochemicals	

1	1
2	1
3	0.8
4	0.8
5	1
6	1
7	1
Occupation	
1	0.8
2	1
3	1
OTC Medications	
1	1
2	1
3	1
4	1
Drinking water	
1	1
2	1
3	1
4	1
5	1
6	0.8
Heat stress	
1	1
2	1
Air pollution	
1	1
2	1
3	1
4	1
5	1

Worm infestations	
1	0.8
2	1
3	1
Food and nutrition	
1	1
2	1
3	1
4	1
5	1
6	1
7	1
8	1

S-CVI : 0.98

b. Appropriateness

Question number	Item content validity
Personal information	
1	1
2	0.8
3	1
4	1
5	1
6	1
7	1
8	1
9	1
10	1
11	1
12	1
13	1
14	1

Past health issues	
1	1
2	1
3	1
4	1
5	1
6	0.8
7	0.8
8	0.8
9	0.8
10	0.8
11	1
12	1
Agrochemicals	
1	1
2	1
3	0.8
4	0.8
5	0.8
6	0.8
7	1
Occupation	
1	0.6
2	1
3	0.8
OTC Medications	
1	0.8
2	1
3	1
4	1
Drinking water	
1	1

2	1
3	1
4	1
5	1
6	1
Heat stress	
1	0.8
2	1
Air pollution	
1	1
2	1
3	1
4	0.8
5	1
Worm infestations	
1	0.8
2	1
3	1
Food and nutrition	
1	1
2	1
3	1
4	0.8
5	1
6	1
7	1
8	1

S-CVI: 0.98

c. Relevance

Question number	Item content validity
Personal information	

1	1
2	0.6
3	1
4	1
5	1
6	1
7	1
8	1
9	1
10	1
11	1
12	1
13	1
14	1
Past health issues	
1	1
2	1
3	0.8
4	0.8
5	1
6	1
7	1
8	0.8
9	1
10	0.8
11	0.8
12	1
Agrochemicals	
1	1
2	1
3	0.8
4	0.8

5	1
6	1
7	1
Occupation	
1	0.8
2	1
3	1
OTC Medications	
1	1
2	1
3	1
4	1
Drinking water	
1	1
2	1
3	1
4	1
5	1
6	1
Heat stress	
1	1
2	1
Air pollution	
1	1
2	1
3	1
4	1
5	1
Worm infestations	
1	0.8
2	1
3	1

Food and nutrition	
1	1
2	1
3	1
4	1
5	1
6	1
7	1
8	1

S-CVI: 0.98

Supplementary file SIV: References

27. Nandakumar UP, Joel JJ, Shetty J, et al (2021) Preparation, validation and user-testing of patient information leaflet on cancer. Pharm Hosp Clin 56:12–17. https://doi.org/10.1016/j.phclin.2020.07.012

28. Rodrigues IB, Adachi JD, Beattie KA, MacDermid JC (2017) Development and validation of a new tool to measure the facilitators, barriers and preferences to exercise in people with osteoporosis. BMC Musculoskelet Disord 18:540. https://doi.org/10.1186/s12891-017-1914-5

29. Windsor R (2015) Evaluation of Health Promotion and Disease Prevention Programs: Improving Population Health through Evidence-Based Practice. Oxford University Press, Oxford, New York

30. Unit of Biostatistics and Research Methodology, School of Medical Sciences, Universiti Sains Malaysia, Kelantan, MALAYSIA, Arifin WN (2018) A Web-based Sample Size Calculator for Reliability Studies. Educ Med J 10:67–76. https://doi.org/10.21315/eimj2018.10.3.8

31. Frost MH, Reeve BB, Liepa AM, et al (2007) What Is Sufficient Evidence for the Reliability and Validity of Patient-Reported Outcome Measures? Value Health 10:S94–S105. https://doi.org/10.1111/j.1524-4733.2007.00272.x

32. Hitchon CA, Zhang L, Peschken CA, et al (2020) Validity and Reliability of Screening Measures for Depression and Anxiety Disorders in Rheumatoid Arthritis. Arthritis Care Res 72:1130–1139. https://doi.org/10.1002/acr.24011

33. IBM Corp (2022) IBM SPSS Statistics for Windows

34. Altman DG (1990) Practical Statistics for Medical Research. CRC Press

35. McHugh ML (2012) Interrater reliability: the kappa statistic. Biochem Medica 22:276–282

36. Cicchetti DV (1994) Guidelines, criteria, and rules of thumb for evaluating normed and standardized assessment instruments in psychology. Psychol Assess 6:284–290. https://doi.org/10.1037/1040-3590.6.4.284

37. Nerbass FB, Pecoits-Filho R (2019) Can your work affect your kidney's health? Rev Environ Health 34:441–446. https://doi.org/10.1515/reveh-2019-0014

38. Dhindsa DS, Khambhati J, Schultz WM, et al (2020) Marital status and outcomes in patients with cardiovascular disease. Trends Cardiovasc Med 30:215–220. https://doi.org/10.1016/j.tcm.2019.05.012

39. Campbell S, Soman-Faulkner K (2025) Antiparasitic Drugs. In: StatPearls. StatPearls Publishing, Treasure Island (FL)

40. Paidi G, Iroshani Jayarathna AI, Salibindla DBAMR, et al Chronic Kidney Disease of Unknown Origin: A Mysterious Epidemic. Cureus 13:e17132. https://doi.org/10.7759/cureus.17132

41. Rubio DM, Berg-Weger M, Tebb SS, et al (2003) Objectifying content validity: Conducting a content validity study in social work research. Soc Work Res 27:94–104. https://doi.org/10.1093/swr/27.2.94

42. Lynn MR (1986) Determination and quantification of content validity. Nurs Res 35:382–385

43. Lawshe CH (1975) A Quantitative Approach to Content Validity1. Pers Psychol 28:563–575. https://doi.org/10.1111/j.1744-6570.1975.tb01393.x

44. Marx RG, Menezes A, Horovitz L, et al (2003) A comparison of two time intervals for test-retest reliability of health status instruments. J Clin Epidemiol 56:730–735. https://doi.org/10.1016/s0895-4356(03)00084-2

45. Beaton DE, Boers M, Tugwell P (2017) Chapter 33 - Assessment of Health Outcomes. In: Firestein GS, Budd RC, Gabriel SE, et al (eds) Kelley and Firestein's Textbook of Rheumatology (Tenth Edition). Elsevier, pp 496–508

46. Park MS, Kang KJ, Jang SJ, et al (2018) Evaluating test-retest reliability in patient-reported outcome measures for older people: A systematic review. Int J Nurs Stud 79:58–69. https://doi.org/10.1016/j.ijnurstu.2017.11.003

47. Heale R, Twycross A (2015) Validity and reliability in quantitative studies. Evid Based Nurs 18:66–67. https://doi.org/10.1136/eb-2015-102129

48. Gravesande J, Richardson J, Griffith L, Scott F (2019) Test-retest reliability, internal consistency, construct validity and factor structure of a falls risk perception questionnaire in older adults with type 2 diabetes mellitus: a prospective cohort study. Arch Physiother 9:14. https://doi.org/10.1186/s40945-019-0065-4

49. Johnson JL, Adkins D, Chauvin S (2020) A Review of the Quality Indicators of Rigor in Qualitative Research. Am J Pharm Educ 84:7120. https://doi.org/10.5688/ajpe7120

50. Health I of M (US) C for the S of the F of P (1988) Public Health as a Problem-Solving Activity: Barriers to Effective Action. In: The Future of Public Health. National Academies Press (US)
